# Enzyme-Assisted Photoinjection of Megadalton Molecules into Intact Plant Cells Using Femtosecond Laser Amplifier

**DOI:** 10.1038/s41598-019-54124-y

**Published:** 2019-11-26

**Authors:** Taufiq Indra Rukmana, Gabriela Moran, Rachel Méallet-Renault, Misato Ohtani, Taku Demura, Ryohei Yasukuni, Yoichiroh Hosokawa

**Affiliations:** 1 Division of Materials Science, Graduate School of Science and Technology, Nara Institute of Science and Technology, 630-0192 Ikoma, Japan; 2 Université Paris-Sud, Université Paris-Saclay, Institut des Sciences Moléculaires d’Orsay (ISMO), CNRS, F-91405 Orsay, France; 3 Division of Biological Science, Graduate School of Science and Technology, Nara Institute of Science and Technology, 630-0192 Ikoma, Japan

**Keywords:** Gene delivery, Plant biotechnology, Optical techniques

## Abstract

Femtosecond laser photoporation has become a popular method to deliver various kinds of molecules such as genes, proteins, and fluorescent dyes into single mammalian cells. However, this method is not easily applied to plant cells because their cell wall and turgor pressure prevent the delivery, especially for larger molecules than the mesh size of the cell wall. This work is the first demonstration of the efficient photoinjection of megadalton molecules into a cytoplasm of an intact single plant cell by employing a femtosecond laser amplifier under moderate enzyme treatment conditions. The intense femtosecond laser pulse effectively formed a pore on the cell wall and membrane of Tobacco BY-2, and 2 MDa dextran molecules were introduced through the pore. Along with the pore formation, induced mechanical tensile stresses on BY-2 cells were considered to increase permeability of the cell membrane and enhance the uptake of large molecules. Moreover, the moderate enzyme treatment partially degraded the cell wall thereby facilitating the increase of the molecular introduction efficiency.

## Introduction

Gene introduction techniques to plant cells have become increasingly important for molecular breeding with the recent progress in plant genome sequencing^1^. The main goals of molecular breeding of plants include the addition of superior properties to agricultural crops such as a high yielding ability and disease and stress resistances^2^. Production of useful substances, including drugs and antibodies, and environmental purification through genetically modified plants are also active fields in plant research^3–5^. In fundamental plant physiological studies, as well, gene introduction is an indispensable tool to understand individual gene functions and to design plant functions for the above applications^5–8^.

Among several methods to introduce foreign genes into plants, polyethylene glycol and electroporation methods need preparation of protoplasts which are plant cells whose cell wall has been removed^9,10^. The use of protoplasts demands careful handling and is time consuming due to laborious protocols; thus methods requiring protoplasts are infrequently used nowadays^1,2,5^. Instead, agrobacterium and particle gun-based methods are practically carried out. However, the use of agrobacterium has an intrinsic safety problem because of unexpected gene modifications^1,11^. The particle gun is a safe technique and applicable to a large variety of plants, although it also faces the problem of fragmentation of introduced DNA^1,2,5,12^.

As an alternative gene transfer method to intact plant cells, laser photoinjection is a possible candidate. In a general protocol for laser photoinjection to plants, a pulsed laser is focused onto a cell to induce a transient pore formation for simple introduction of exogenous substances into cells. This process is done in a hypertonic solution to reduce plant turgor pressure which originates from the inner pressure of growth vacuoles in a plant cell. Weber *et al*.^13,14^ applied a UV nanosecond laser to perforate a cell wall in a buffer containing 0.4 M of sorbitol, and they introduced DNA plasmid into plant cells of *Brassica napus* for the first time in 1988. Following their work, in 1995, Guo *et al*.^15^ confirmed the expression of transferred gene in rice cells with a UV nanosecond Nd:YAG laser while gene expression was seen in only 0.48% of cells. In addition, the intense UV laser light has a risk of causing photochemical damage in its optical path, consequently compromising cell viability and ability to express the transferred gene.

More recently, a near infrared femtosecond (fs) laser has been employed for the nanoprocessing and photoinjection to plants^16–18^. One of the main advantages of the fs laser photoinjection is the precise perforation capability at a subcellular resolution thanks to effective non-linear processes such as a multi-photon absorption at a laser focal point, which suppresses collateral damage in the optical path. Indeed, an efficient gene transfection by the fs laser photoinjection has already been reported for mammalian cells^19–24^. However, injection of large molecules to plant cells is much more difficult compared with that to animal cells. Using fs laser photoinjection, LeBlanc *et al*.^17^ introduced fluorescein isothiocyanate-conjugated dextran (FITC-dextran) of 10 kDa into an intact single cell of *Arabidopsis thaliana*. Mitchell *et al*.^18^ reported a systematic study of fs laser photoinjection efficiency of FITC-dextran into a tobacco BY-2 cell (TBY-2) considering the dependence on osmolarity, laser power and molecular weight of the conjugated dextran. They concluded that the introduction efficiency was significantly reduced with increasing molecular weight, and the maximum size of introduced dextran was 40 kDa under optimized conditions.

As it stands now, for larger molecules like DNA plasmids whose molecular weights are mostly over 1 MDa, their introduction into intact plant cells with fs laser photoinjection has not been achieved yet to the best of our knowledge. One conceivable limitation would be that a plant cell membrane is protected by a relatively thick and rigid cell wall consisting of a cellulose fiber network, and only substances with a diameter smaller than around 30 kDa can pass through this network. Besides, in the fs laser photoinjection experiments referred to above, their results implied that the effective perforated point is very small or the perforation occurs only on the cell membrane. This processing property could be due to application of fs laser pulses with a low pulse energy (<1 nJ/pulse) and high repetition rate (>10 MHz)^18^. This type of fs laser is widely used for two-photon imaging in biology. Therefore, from an instrumental aspect, it is convenient to use the same system for fs laser photoinjection. Meanwhile, because of its high repetition rate, increasing of the laser pulse energy causes accumulation of thermal energy and formation of a long-lasting vapor bubble at a laser focal point. The pore formation of the rigid cell wall is likely to compensate to a decrease of cell viability. For these reasons, the cell wall would still be a barrier for the diffusion of large substances into a cytosol even though a small pore was formed on it by the fs laser photoporation.

In this work, we demonstrated introduction of FITC conjugated dextran of 20 kDa (FITC-20k) and of 2 MDa (FITC-2M) into TBY-2 with a fs Ti:sapphire laser amplifier under a moderate enzyme treatment with cellulase and pectolyase which degrade main components of the cell wall. The use of intense fs laser pulses is expected to perforate the cell wall efficiently with less thermal damage and the enzyme treatment enhances diffusion of large molecules through the cell wall without generating a protoplast by its partial degradation.

## Results

### Effect of mannitol addition and enzyme treatment on cell structures

**Figure 1:**
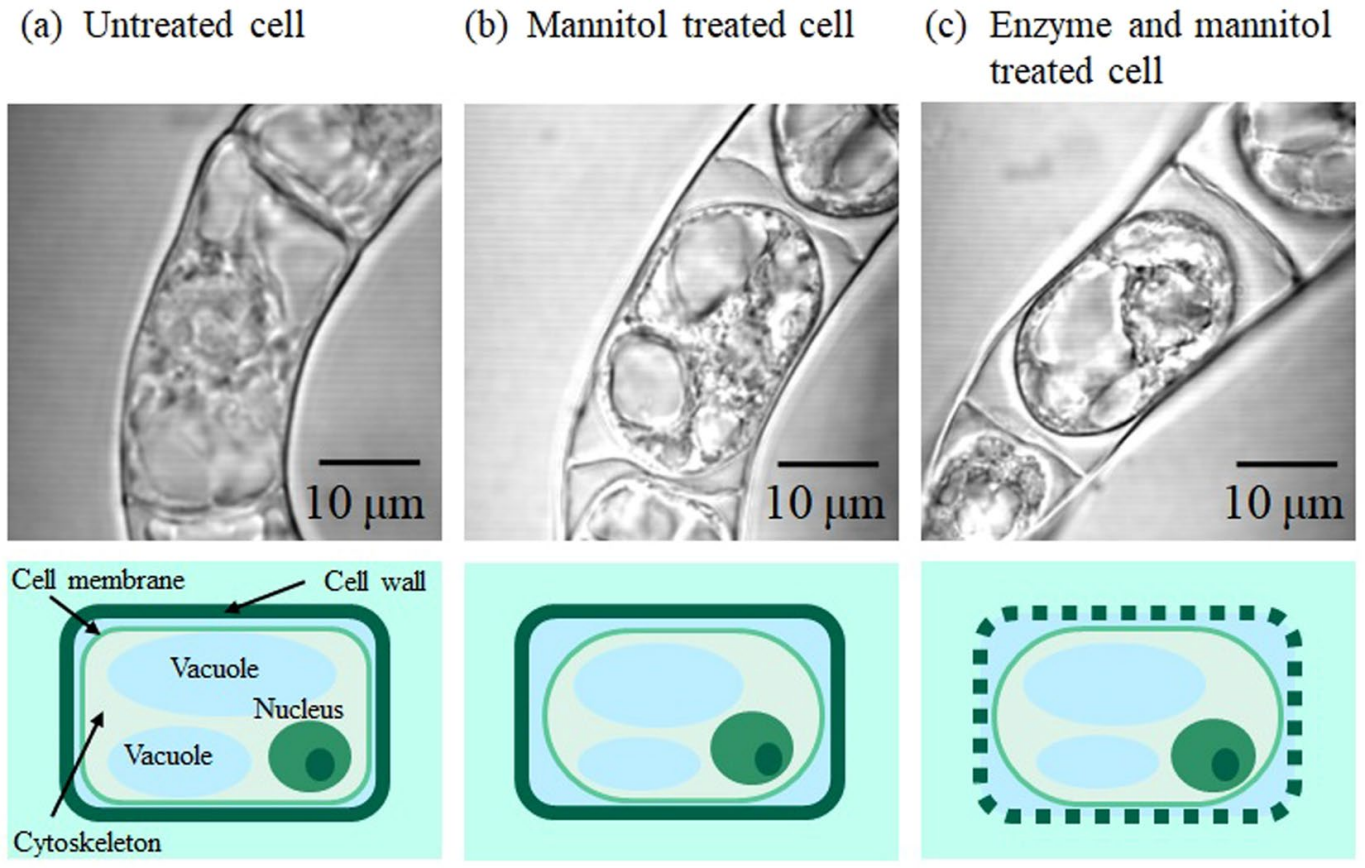
Transmission images and their schematic structures of TBY-2 cells; (**a**) untreated, (**b**) mannitol treated (plasmolyzed) and (**c**) adjunctive enzyme treated (enzyme and mannitol treated). In an untreated TBY-2 cell, organelles such as a nucleus, plastids, vacuoles and a cytoskeleton are inside the cell membrane. The cell membrane is covered with a cell wall consisting of polysaccharides such as cellulose and pectin. A turgor pressure, which is an inner pressure of the vacuoles observed at corners, presses the cell wall from inside to keep the cell structure and stiffness.

In advance of the fs laser photoporation, we examined a morphological change of intracellular structures of TBY-2 cells (Fig. 1a) after the mannitol addition and enzyme treatments. When mannitol was added to the culture medium, the vacuoles shrunk, and the cell membrane was separated from the cell wall (Fig. 1b), which means that the turgor pressure decreases and the cell is plasmolyzed in the hypertonic mannitol solution. To enhance permeability of the cell wall for larger molecules, we performed the enzyme treatment adjunctively with the mannitol addition. As a general procedure for protoplast preparation, a cell wall is removed by using about a 1 h enzyme treatment. On the other hand, we did not observe a noticeable morphological change for the cell 10 min after the adjunctive enzyme treatment (Fig. 1c). In this moderate process, the cellulose fiber network in the cell wall would not be removed, but degraded partially. The fs laser photoinjection was performed under these three conditions of the cells: untreated, mannitol treated (plasmolyzed) and adjunctive enzyme treated (enzyme and mannitol treated).

### Transient morphological change induced by the fs laser irradiation

When a near-infra-red (NIR) fs laser pulse is focused on the cell wall and membrane of the TBY-2 cells through a high numerical aperture (NA) objective lens, efficient non-linear processes represented by the multiphoton absorption lead to a laser ablation of these components at the laser focal point. In addition, the ablation induces rapid expansion of a cavitation bubble and a tensile stress wave propagates to the periphery. These phenomena mechanically act on the cell wall and membrane. These mechanical phenomena are especially significant in amplified intense laser pulses: the higher energy density at the laser focal point causes the higher stress increase^25^. In this context, it is valuable to verify the mechanical effects on TBY-2 morphology in the photoinjection process with the fs laser amplifier.

**Figure 2:**
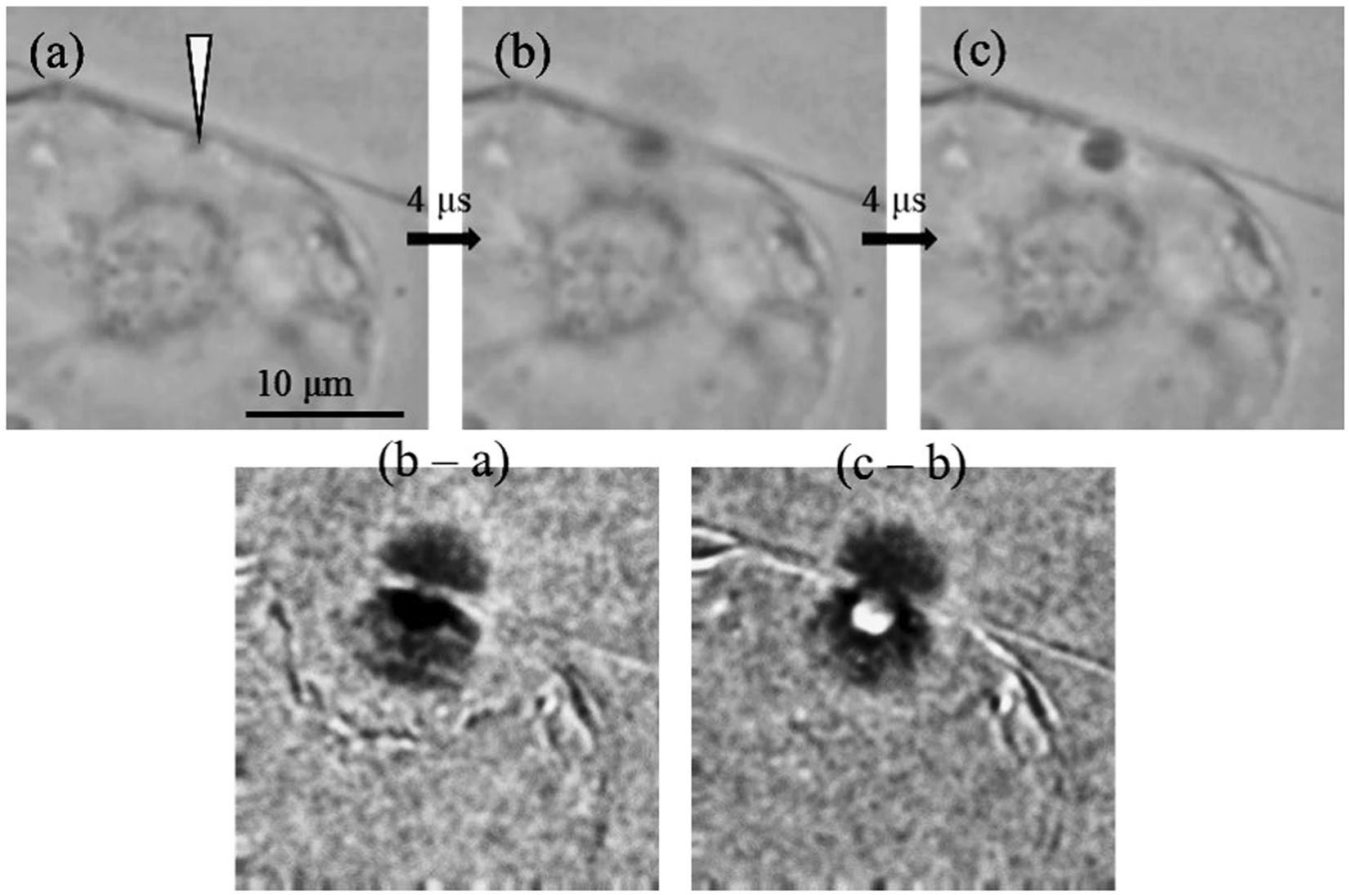
A series of high-speed images (frame rate, 4 μs) taken for the fs laser photoporation experiment. The images (**a**) before laser irradiation, (**b**) 4 μs after irradiation and (**c**) 8 μs after it. The laser focal point is indicated with the white arrow. The differential images were obtained by image calculation of Image J.

Representative results of the transient morphological change of TBY-2 that we acquired with a high-speed camera are shown in Fig. 2. When the fs laser pulse with an energy of 20 nJ was focused on the cell wall, generation of the cavitation bubble was observed. Differential images revealed instantaneous deformation of the cell membrane and wall of the TBY-2 cell around the laser focal point, and that deformation reverted to the initial state in about 8 μs. A gas bubble also appeared at the laser focal point but it remained at most for only a few milliseconds.

### Molecular introduction in the hypertonic solution

**Figure 3:**
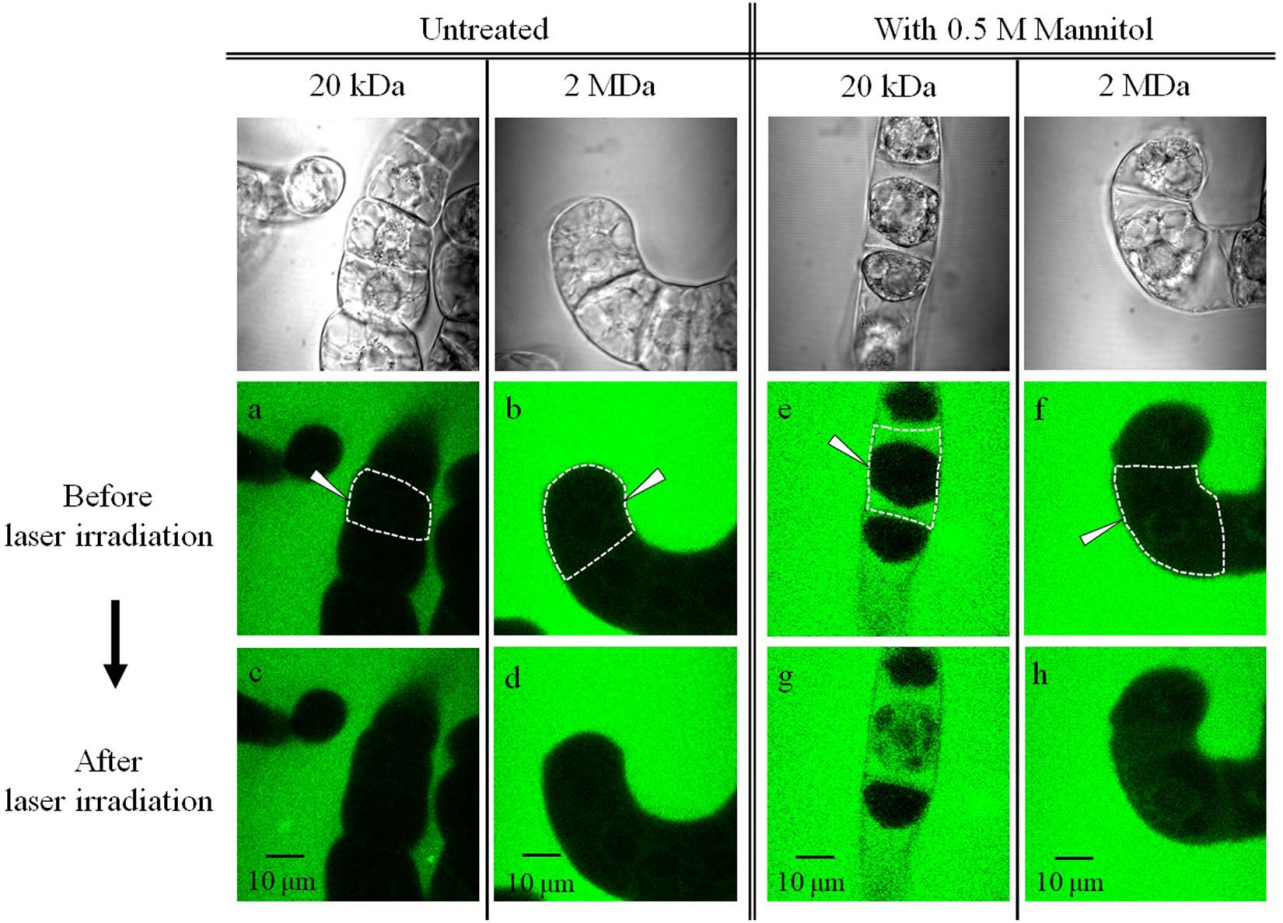
Confocal fluorescence images of (**a**–**d**) untreated and (**e**–**h**) mannitol added plasmolyzed TBY-2 cells before and after fs laser irradiation under the presence of FITC-20k and FITC-2M. Corresponding transmission images before laser irradiation were shown on the top row to identify each cell. The target single cells are surrounded by a dashed line, and the focal point is indicated with a white arrow. These images express the representative fluorescence intensity change that (**a**–**d**) no difference, (**e**,**g**) large increase and (**f**,**h**) slight increase in their appearance. The excitation and emission maxima of FITC are 490 and 520 nm, respectively. All fluorescence images were acquired with the excitation laser at 488 nm and same PMT gains.

We first examined photoinjection efficiencies of FITC-20k and of FITC-2M into untreated TBY-2 cells without any treatment, and into plasmolyzed TBY-2 cells in the hypertonic solution of 0.5 M mannitol. Before and 2 min after fs laser irradiation, fluorescence images of the cross section of target cells were captured with a confocal fluorescence microscope. Figure 3(a,b) show representative fluorescence images of untreated TBY-2 cells before fs laser irradiation under the presence of FITC-20k and FITC-2M in the culture medium. Fluorescence was not observed inside the cell for both sizes of FITC-dextran molecules confirming that those molecules did not enter into the cells spontaneously. After the mannitol treatment, plasmolysis was clearly seen for FITC-20k and FITC-2M from their transmission images in Fig. 3. The fluorescence signal was observed within apoplast where in between the cell wall and membrane only for FITC-20k but not FITC-2M as seen in Fig. 3(e,f). This result indicates that FITC-20k is able to pass through the cell wall but not the cell membrane, which is in good agreement with the previous report that smaller molecules than 30 kDa (hydrodynamic diameter of about 5 nm) can cross the cell wall^14^. Meanwhile, FITC-2M is too large and still unable to cross the cell wall.

Next, we focused a single fs laser pulse on the cell at a contact point of the cell membrane and cell wall in the presence of FITC-dextran molecules. Fluorescence images 2 min after the laser irradiation for untreated and plasmolyzed cells are shown in Fig. 3(c,d,g,h). These images were the case in which the most frequently observed for each condition. The fluorescence images had almost no change before and after laser irradiation for the untreated cells. In contrast, the increase of fluorescence intensity within cytoplasm was observed for both FITC-20k and FITC-2 M for the plasmolyzed cells. Also, no increase of auto-fluorescence was detected under the same photoporation condition without FITC molecules in the culture medium. Therefore, we concluded that FITC molecules were successfully injected into the cytoplasm. In this experiment, we examined pulse energies with 10, 20 and 30 nJ to investigate the photoinjection efficiencies (Fig. S1). The injection efficiency was very low with 10 nJ/pulse, and no significant difference between with 20 and 30 nJ/pulse under the enzyme treated condition. In the meantime, cells started getting damage with 30 nJ/pulse. Therefore, the pulse energy of 20 nJ was selected for the experiment.

In order to clarify the effect of fs laser irradiation on the cell membrane and cell wall, photoinjection was performed by focusing the fs laser pulse specifically on the cell membrane without irradiating the cell wall for the plasmolyzed cells. Fluorescence increases inside the cytoplasm were observed only for FITC-20k (Fig. S2). This indicates that the pore formation only on the cell membrane is necessary to introduce FITC-20k because molecules are already present within apoplast under the mannitol treatment. On the other hand, as FITC-2 M is blocked by the cell wall, the pore formation not only on the cell membrane but also the cell wall is required for the injection of FITC-2 M into the cell membrane.

### Molecular introduction with enzyme assistance

In the previous section, we stated that the pore formation on the cell membrane at the presence of FITC-molecules within apoplast was a critical factor for their injection into the cytoplasm. However, permeability increase of the cell wall through the pore formed by the fs laser irradiation was not very efficient for FITC-2M molecules. In order to increase permeability of the cell wall for large molecules with the minimum damage, enzyme treatment with cellulase and pectolyase was applied for degrading the cell wall.

**Figure 4:**
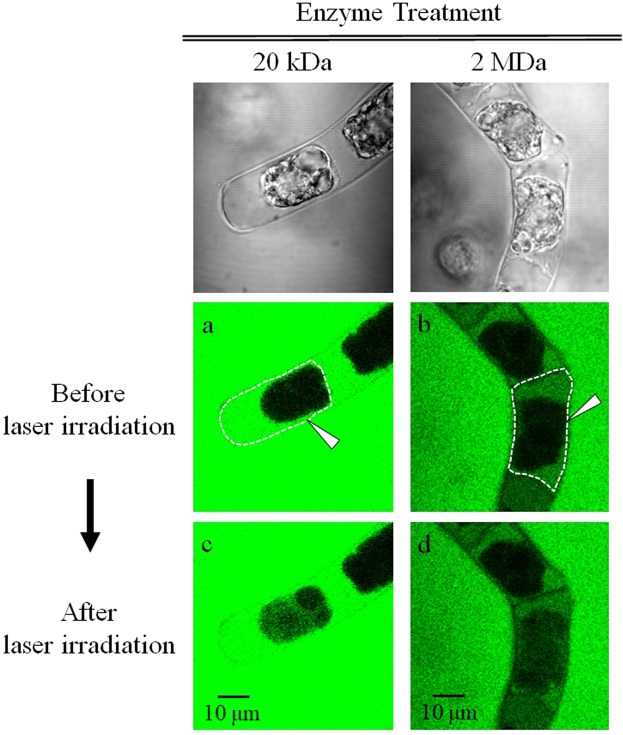
Confocal fluorescence images of adjunctive enzyme treated TBY-2 cells before and after fs laser irradiation under presence of FITC-20k and FITC-2M. Corresponding transmission images before laser irradiation were shown on the top row to clarify each cell. The target single cells are surrounded by a dashed line, and the focal point is indicated with a white arrow. The fluorescence images were acquired with the same conditions as those in Fig. 3.

Representative fluorescence images of adjunctive enzyme treated TBY-2 cells before the photoporation in the presence of FITC molecules are shown in Fig. 4(a,b). Unlike the results shown in Fig. 3(b,f), fluorescence was detected within apoplast even for FITC-2M. Under this condition, the permeability of the cell membrane seemed to be unchanged. Indeed, it was always dark inside the cell membrane.

Regarding the adjunctive enzyme treated TBY-2 cells in Fig. 4(a,b), fluorescence images 2 min after the laser irradiation are shown in Fig. 4(c,d). The fluorescence intensity increase was clearly observed within cytoplasm for both FITC-20k and FITC-2M. A noteworthy point is that the fluorescence signal increase was enhanced compared with only the mannitol addition not just by the adjunctive enzyme treatment as described in detail later. Cytoplasmic streaming was seen and thus there was no significant damage to the cell viability.

### Introduction efficiencies under three conditions

**Figure 5:**
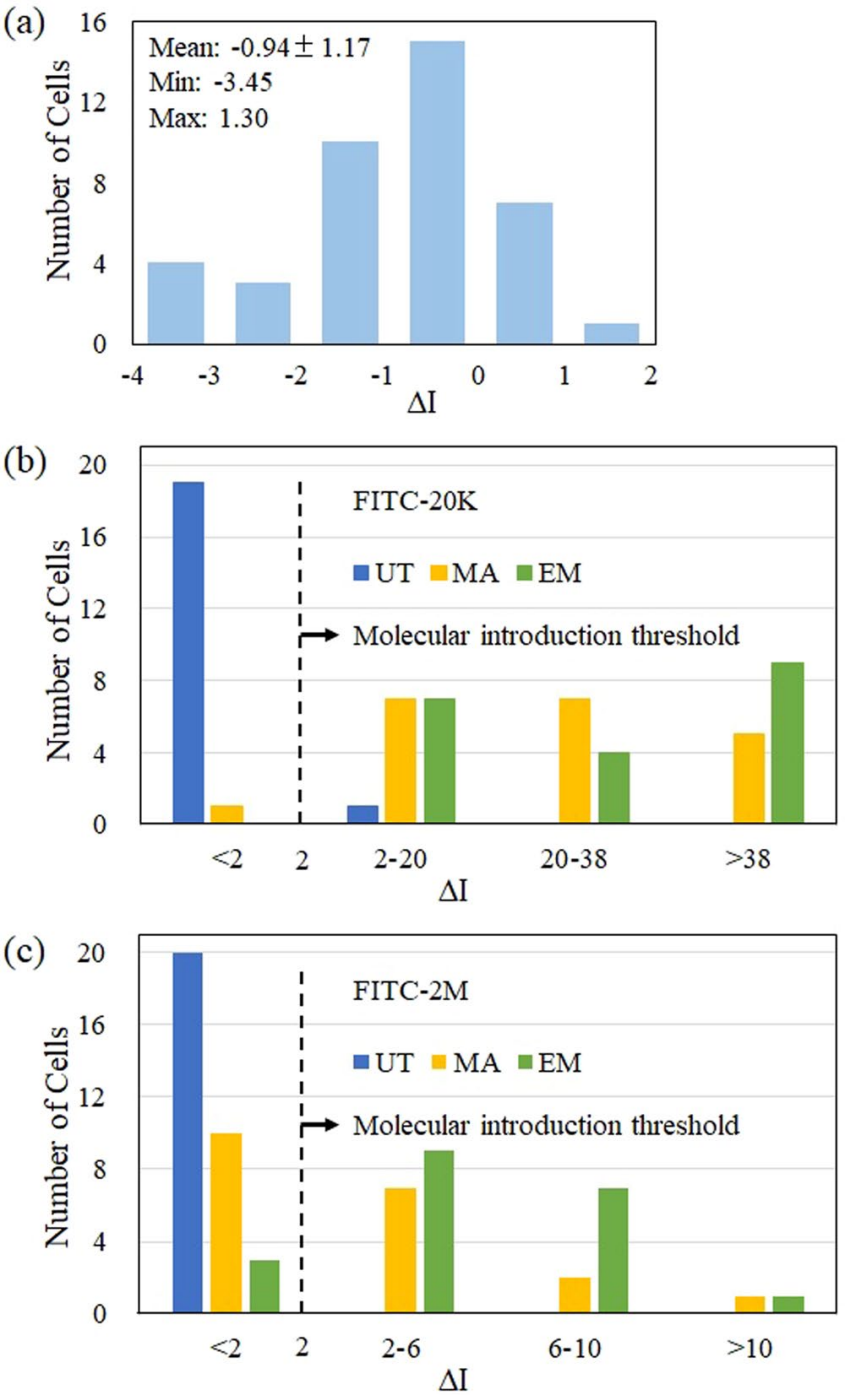
(**a**) The histograms of intensity fluctuations of the background signal inside the cytoplasm during 2 min without laser irradiation (N = 40). The histograms of differential fluorescence intensities (*∆I*) between before and after laser irradiations of untreated (UT, blue), mannitol treated (MA, yellow) and adjunctive enzyme treatment (EM, green) cells: (**b**) for FITC-20k (N = 20) and (**c**) for FITC-2M (N = 20).

The level of introduced FITC molecules under the three conditions, untreated, mannitol treated and adjunctive enzyme treated, were quantified by differential fluorescence intensities measured in cytosol between before and after the photoporation (*∆I*). Even before laser irradiations, there was a small background fluorescence signal inside the cytoplasm that originated from cell’s auto fluorescence and/or scattered fluorescence by cells. In Fig. 5(a), background fluorescence intensity changes during 2 min without laser irradiation are shown as a histogram. The temporal fluctuations of the background fluorescence for 40 cells varied from -3.45 to 1.30 with a mean of -0.94. The negative values were due to fluorescence bleaching during the laser scanning. From the background temporal fluctuations estimation, we define *∆I* as molecular introduction level and the *∆I* value of 2 is called the molecular introduction threshold. The *∆I* values for 20 cells for each condition are summarized as histograms in Fig. 5(b,c). The representative observations in Figs. 3 and 4 were well reflected in the present histograms as told below.

The average values of *∆I* increased in the order of untreated cells, plasmolyzed cells and adjunctive enzyme treated cells. For the untreated cells, most *∆I* values were smaller than 2 (the introduction threshold) for both FITC-20k and FITC-2M, indicating FITC molecules were not introduced into the cells by the photoporation in a hypotonic solution. On the other hand, for the plasmolyzed cells after the mannitol addition, the *∆I* distribution of both FITC-20k and FITC-2M shifted to a higher level than the background fluctuations. This result demonstrates that both sizes of FITC molecules are successfully introduced into the cytosol by the photoporation in the hypertonic condition. We also measured the temporal changes of *∆I* after the photoporation. *∆I* reached to the maximum in about 30 s for FITC-20k, reflecting its rapid diffusion in cytoplasm. From the time to reach maximum *∆I* for FITC-2M whose diffusion would be much slower than FITC-20k, we estimate that the formed pore is resealed roughly in 100 to 150 s (Fig. S3).

Although a leakage of cell contents, which is a sign of cell damage by the laser irradiation, was observed for 25% of the untreated cells in transmission images (Fig. S4), such outflow was not confirmed for any plasmolyzed cells. Reduction of the turgor pressure presumably minimizes the cell damage by preventing leakage of the cytosol. In fact, cytoplasmic streaming was observed in the plasmolyzed cells even after the photoporation. These results indicate the cell is still viable after the photoporation. However, the introduction efficiency for FITC-2M was not very high as half the *∆I* values were still close to the background level.

**Figure 6:**
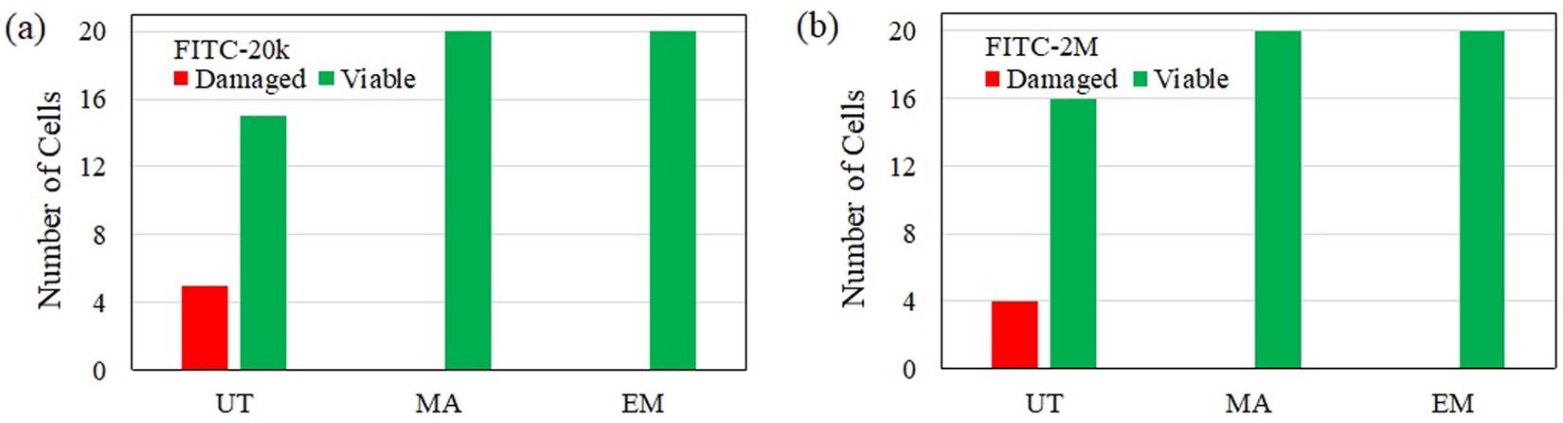
The number of damaged (red) and viable (green) cells after laser irradiation in untreated (UT), mannitol treatment (MA) and adjunctive enzyme treatment (EM) with laser energy of 20 nJ/pulse: (**a**) FITC-20k and (**b**) FITC-2M.

The adjunctive enzyme treatment led to further shift of the *∆I* distribution of both FITC-20k and FITC-2 M to a higher level than the plasmolyzed cells. The typical increase of *∆I* was relatively larger for FITC-2M than that for FITC-20k. In this condition, the cytoplasmic streaming was also seen in all cells after the photoporation, therefore we regard the enzyme treatment as not augmenting damage of the photoinjection process. The number of damaged cells with laser treatment in each condition was summarized in Fig. 6.

## Discussion

We could introduce FITC-20k and -2M into TBY-2 cells by fs laser photoporation in the hypertonic condition by mannitol addition (Figs. 3 and 5). The enzyme treatment enhances the introduction efficiency especially for FITC-2M (Figs. 4 and 5). The effects of osmolarity are in good agreement with previously reported findings on fs laser photoinjection into plant cells which showed the introduction of dye-conjugated dextran molecules was achieved only in a hypertonic solution. However, the sizes of introduced molecules were limited to only a few tens of kDa in these experiments in contrast to our results of 2 MDa. Our success for the larger introduced molecular size would be explained by the different type of fs laser system we used for the perforation. We used the single-shot laser pulse with the energy of 20 nJ from the fs laser amplifier whereas the reported studies used low-energy and high-repetition rate pulses from an fs laser oscillator. The much higher laser fluence in our experiments would induce an efficient perforation of the cell wall and the cell membrane at the laser focal point with less heat accumulation. This assumption is also supported by the report for mammalian cells that single higher energy fs laser pulse can form a twice larger pore compared with trains of low energy pulses in optimal conditions on photoinjection efficiency and cell viability for these irradiation regimes^22^. Moreover, generated tensile stress around the pore should be stronger for the fs laser amplifier than the fs laser oscillator^25^. In Fig. 2, the transient morphological change by the laser-induced mechanical stress was observed for the TBY-2 cell. Therefore, we currently consider that the propagation of strong tensile stress associated with the fs laser photoporation may have an important role to increase permeability of the cell membrane instantaneously.

Further increases of *∆I* for both FITC-20k and -2M were achieved after the adjunctive enzyme treatment. From the transmission (Fig. 1) and fluorescence images (Fig. 4), this treatment partially degrades the cell wall network without forming a protoplast, and it contributes to increase of the molecule diffusion rate through the cell wall. As FITC-20k can diffuse without pore formation by the fs laser, the effect of the adjunctive enzyme treatment is more significant for FITC-2M. The high concentration of FITC-2M molecules inside the cell wall permits their introduction into the cytoplasm efficiently. This treatment also softens the cell wall resulting in an increase in the degree of deformation by the mechanical stress. The larger deformation of TBY-2 cells under adjunctive enzyme treatment might contribute to the enhanced photoinjection efficiency by the amplified fs laser pulse.

## Conclusion

This work is the first demonstration of the photoinjection of megadalton molecules into a cytoplasm of an intact single plant cell by employing a femtosecond laser amplifier under moderate enzyme treatment conditions. Our results indicate that application of the photoinjection using the amplified fs laser pulse has good potential for efficient introduction of large molecules to intact plant cells although introduction of actual genes and their expression still must be examined. The precise targeting capability is the most important advantage of the fs laser photoinjection^26^. Developing a transfection technique into an intact single cell of a plant tissue is expected to expand insights into plant systems being used in gene modifications.

## Materials and Methods

### Preparation of Tobacco BY-2 cells

Wild-type TBY-2 cells were cultured as described elsewhere with slight modifications^27,28^. The TBY-2 cells were grown in a suspension of 4.6% (w/v) of Murashige–Skoog (MS) basal medium (Ducheva Biochemie) supplemented with 3% (w/v) of sucrose (Sigma-Aldrich), 0.2 g/L of KH_2_PO_4_ (Sigma-Aldrich), 1 mg/L of thiamine-HCl (Sigma-Aldrich), 100 mg/L of myoinositol (Sigma-Aldrich), and 0.1 mg/L of 2,4-dichlorophenoxyacetic acid (Sigma-Aldrich). The cell suspension was agitated at 130 rpm on a rotary shaker in a dark place at 27 °C. The cells were subcultured at weekly intervals by transferring 2 mL of the suspension into 95 mL of the fresh medium.

Two-day old cultured cells were used for the photoporation experiments. First, 1 mL of the cell suspension was centrifuged at 1000 rpm for 1 min, and a pellet of TBY-2 cells was re-suspended in 1 mL of the same culture medium. A mannitol treatment was done to control osmotic pressure by adding 0.5 M of mannitol into the final culture medium.

Enzyme treatment was applied to partially degrade the cell wall. The standard culture medium was replaced with 1 mL of an enzyme solution after the centrifugation. The enzyme solution was prepared as 0.05 M of MES buffer (2-(N-morpholino)ethanesulfonic acid) (Sigma-Aldrich) with a pH of 5.8 containing 0.5% (w/v) of cellulase Onozuka RS (Serva Electrophoresis), 0.05% (w/v) of pectolyase Y-23 (Serva Electrophoresis), and 0.3 M of mannitol (Sigma-Aldrich). The cells were incubated in the enzyme solution in a dark place at 27 °C for 10 min, washed 5 times with 1 mL of 0.4 M of mannitol solution, and then re-suspended in 1 mL of the same culture medium containing 0.5 M mannitol.

Finally, 100 μL of the prepared cell suspensions in each treatment were put in a glass-bottom dish (φ = 12 mm, Iwaki). All the experiments were conducted within 6 hours after each treatment to ensure a healthy condition of the cells was maintained.

### NIR fs laser photoporation

**Figure 7:**
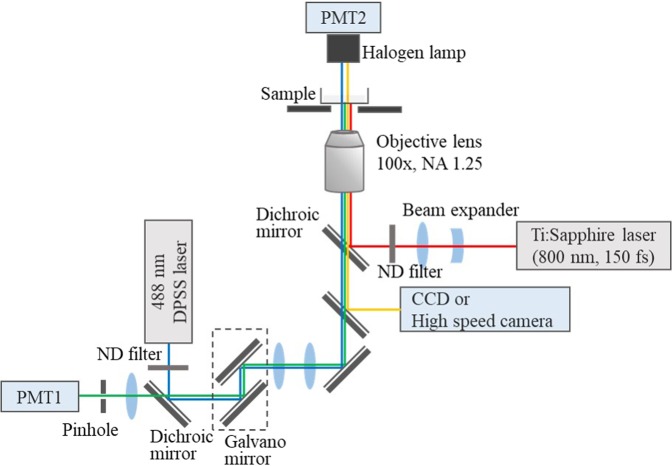
Schematic illustration of the NIR-femtosecond laser system coupled with a confocal laser scanning microscope.

The setup for the photoporation is shown schematically in Fig. 7. Amplified fs laser pulses from a regeneratively amplified fs Ti:sapphire laser system (Spectra-Physics, Solstice-Ref-MT5W, 800 nm, 150 fs) were led into a laser-scanning confocal microscope (Olympus, IX71FVSF-2) and focused on a TBY-2 cell through a 100x oil-immersion objective lens (Olympus; Plan N, NA = 1.25). A single laser pulse was extracted using a mechanical shutter with a gate time of 1/125 s from pulse trains of 125 Hz. The laser pulse energy was tuned by a half waveplate, polarizer, and neutral density filter. The laser beam was expanded by a beam expander to fill the back aperture of the objective lens, and the laser focal plane was set to the image plane of the microscope. The energy of the laser pulse through the objective lens was measured with a laser power meter (Ophir, Nova Display-Rohr). A sample was placed on a motorized microscope stage (Sigma Koki, E-65GR) equipped on the microscope.

### Fluorescence measurements

Fluorescein isothiocyanate (FITC-dextran, Sigma-Aldrich) conjugated with 20 kDa (FITC-20k) or 2 MDa (FITC-2M) dextran were used as external molecules to be introduced into TBY-2 cells. FITC is grafted randomly to hydroxyl groups of dextran at a frequency of 0.003 to 0.02 moles of FITC per mole of glucose. The dye solutions were prepared with concentrations of 0.5 mM for 20 kDa and 2 μM for 2 MDa. The excitation and emission maxima of FITC are 490 and 520 nm, respectively.

The introduction of FITC-dextran was monitored by confocal fluorescence imaging. A diode-pumped solid-state (DPSS) laser (Spectra-Physics, PC14763) at a wavelength of 488 nm was used for excitation of FITC-dextran. 1 μL of FITC-dextran solution was added to 100 μL of the TBY-2 cell suspension in the glass-bottom dish, and the sample was placed on the microscope stage. All fluorescence images were taken with the same acquisition parameters.

The level of introduced FITC-dextran was evaluated as a differential fluorescence intensity (*∆I*) between before and after the photoporation. A cytoplasm of TBY-2 cell was selected as a region of interest using Image J software in the obtained fluorescence image, and the mean intensity value before photoporation (*I*_*before*_) was subtracted from that of 2 min after photoporation (*I*_*after*_). When cells were damaged by the fs laser irradiation, they were excluded from the data set and the histogram of *∆I* was prepared using data of 20 viable cells.

## Supplementary information

Supplementary Information
