# Supplementary material for: Enzyme-Assisted Photoinjection of Megadalton Molecules into Intact Plant Cells Using Femtosecond Laser Amplifier
Source: Sci Rep. 2019 Nov 26;9:17530. doi: 10.1038/s41598-019-54124-y (PMC6879609; doi:10.1038/s41598-019-54124-y)
Supplement: Supplementary file 1 — Supplementary Information [file 41598_2019_54124_MOESM1_ESM.pdf]

Enzyme-Assisted Photoinjection of Megadalton Molecules into Intact Plant Cells Using Femtosecond Laser Amplifier

Taufiq Indra Rukmana[1], Gabriela Moran[2], Rachel Méallet-Renault[2], Misato Ohtani[1], Taku Demura[1], Ryohei Yasukuni[1]*, Yoichiroh Hosokawa[1]

[1]Division of Materials Science, Graduate School of Science and Technology, Nara Institute of Science and Technology, 630-0192, Ikoma, Japan
[2]Université Paris-Sud, Université Paris-Saclay, Institut des Sciences Moléculaires d'Orsay (ISMO), CNRS, F-91405 Orsay, France
[3]Division of Biological Science, Graduate School of Science and Technology, Nara Institute of Science and Technology, 630-0192, Ikoma, Japan

*Email: r-yasukuni@ms.naist.jp

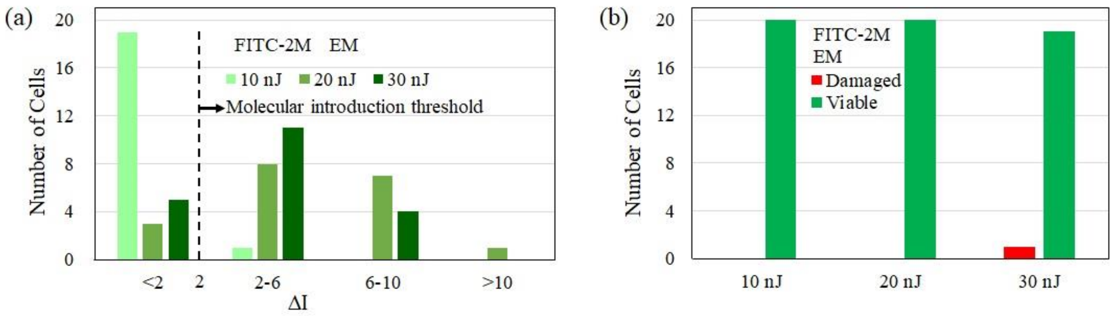

Figure S1 (a) The histograms of differential fluorescence intensities (ΔI) between before and after laser irradiations and (b) the number of damaged (red) and viable (green) cells after laser irradiation of adjunctive enzyme treatment (EM) cells for FITC-2M (N = 20) with laser energy of 10, 20 and 30 nJ/pulse.

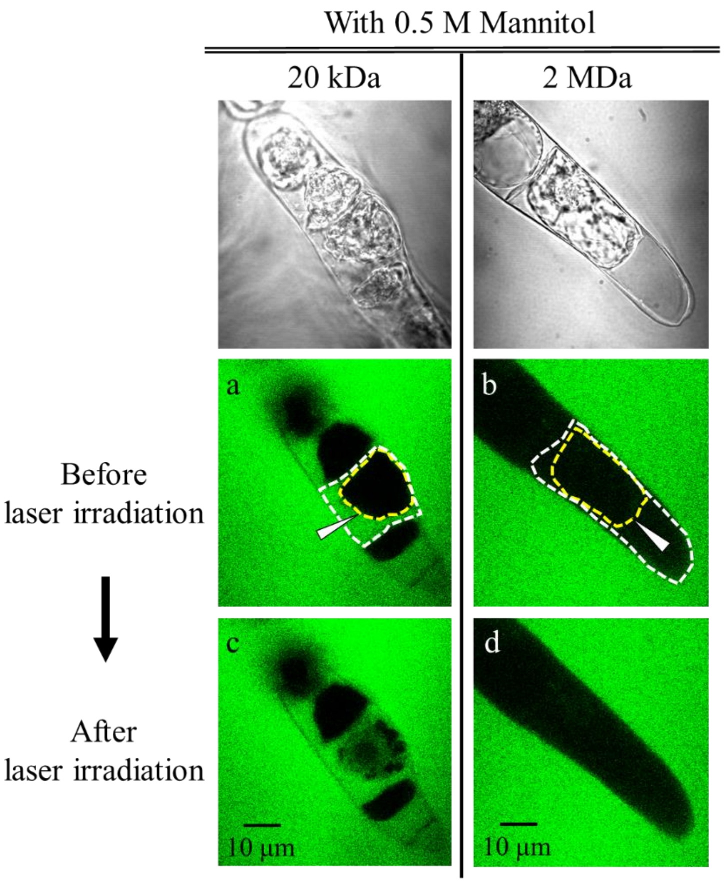

Figure S2 Confocal fluorescence images of mannitol added plasmolyzed TBY-2 cells before and after fs laser irradiation under presence of (a, c) FITC-20k and (b, d) FITC-2M. Corresponding transmission images before laser irradiation were shown on the top row to clarify each cell. The target single cells are surrounded by a white broken line and their cell membranes are surrounded by a yellow dashed line. The fs laser pulse was focused on the cell membrane indicated with the white arrow.

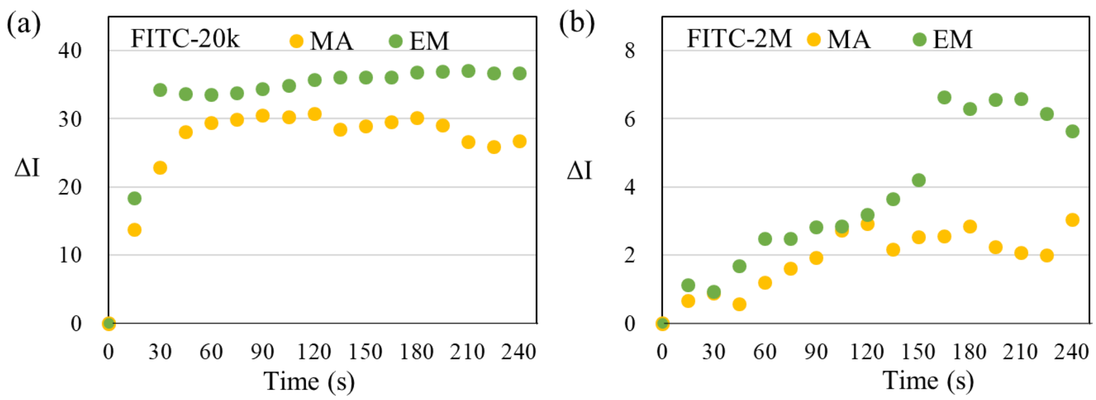

Figure S3 Representative of fluorescence intensity change in cytoplasm over time for mannitol treated (MA, yellow) and adjunctive enzyme treated (enzyme and mannitol treated) (EM, green) cells: (a) for FITC-20k and (b) for FITC-2M.

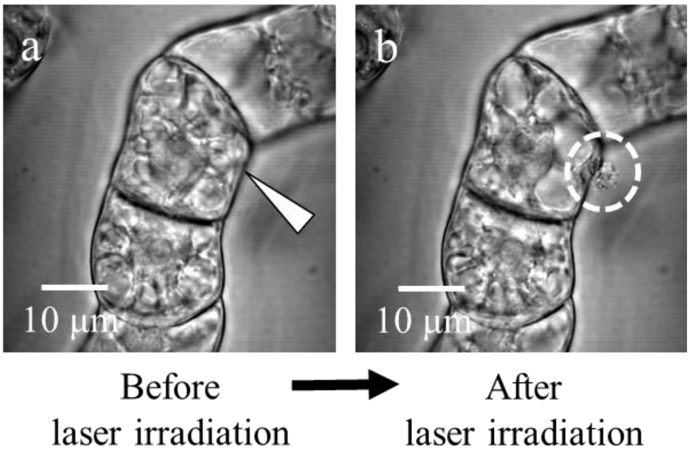

Figure S4 Transmission images of TBY-2 cells (a) before and (b) after the laser irradiation. When cells were damaged, leakage from the cell was observed as seen inside the broken line circle in (b).
